# Search for Strange Quark Matter and Nuclearites on Board the International Space Station (SQM-ISS): A Future Detector to Search for Massive, Non-Relativistic Objects in Space

**DOI:** 10.3390/s24165090

**Published:** 2024-08-06

**Authors:** Massimo Bianchi, Francesca Bisconti, Carl Blaksley, Valerio Bocci, Marco Casolino, Francesco Di Clemente, Alessandro Drago, Christer Fuglesang, Francesco Iacoangeli, Massimiliano Lattanzi, Alessandro Marcelli, Laura Marcelli, Paolo Natoli, Etienne Parizot, Piergiorgio Picozza, Lech Wiktor Piotrowski, Zbigniew Plebaniak, Enzo Reali, Marco Ricci, Alessandro Rizzo, Gabriele Rizzo, Jacek Szabelski

**Affiliations:** 1 Physics Department, Università degli Studi di Roma Tor Vergata, 00133 Rome, Italy; bianchi@roma2.infn.it (M.B.); francesca.bisconti@roma2.infn.it (F.B.); alessandro.marcelli@roma2.infn.it (A.M.); picozza@roma2.infn.it (P.P.); zbigniew.plebaniak@roma2.infn.it (Z.P.); reali@roma2.infn.it (E.R.); 2 RIKEN, Wako 351-0198, Japan; saishokukenbi@gmail.com; 3 INFN (National Institute for Nuclear Physics), Structure of Rome, 00133 Rome, Italy; valerio.bocci@roma1.infn.it (V.B.); francesco.iacoangeli@roma1.infn.it (F.I.); 4 INFN (National Institute for Nuclear Physics), Structure of Rome Tor Vergata, 00133 Rome, Italy; 5 Department of Physics and Earth Sciences, Università degli Studi di Ferrara, 44122 Ferrara, Italy; francesco.diclemente@unife.it (F.D.C.); drago@fe.infn.it (A.D.); paolo.natoli@fe.infn.it (P.N.); 6 KTH Royal Institute of Technology, 11428 Stockholm, Sweden; cfug@kth.se; 7 INFN (National Institute for Nuclear Physics), Structure of Ferrara, 44122 Ferrara, Italy; lattanzi@fe.infn.it; 8 APC (Laboratoire Astroparticule & Cosmologie), Univ Paris Diderot, CNRS/IN2P3, CEA/Irfu, Obs. de Paris, Sorbonne Paris Cité, 75013 Paris, France; parizot@apc.in2p3.fr; 9 Faculty of Physics, University of Warsaw, 00-927 Warsaw, Poland; lech-wiktor.piotrowski@fuw.edu.pl; 10 INFN (National Institute for Nuclear Physics), National Laboratories of Frascati, 00044 Frascati, Italy; marco.ricci@lnf.infn.it; 11 ENEA, Italian National Agency for New Technologies, Energy and Sustainable Economic Development, Radioprotection Institute (IRP), 00196 Rome, Italy; alessandro.rizzo@enea.it; 12 Longviews S.r.l., 00134 Rome, Italy; gabrief@gmail.com; 13 Stefan Batory Academy of Applied Sciences, Stefana Batorego 64C, 96-100 Skierniewice, Poland; jxszabel@gmail.com

**Keywords:** strange quark matter, SQM, ISS, space detector

## Abstract

SQM-ISS is a detector that will search from the International Space Station for massive particles possibly present among the cosmic rays. Among them, we mention strange quark matter, Q-Balls, lumps of fermionic exotic compact stars, Primordial Black Holes, mirror matter, Fermi balls, etc. These compact, dense objects would be much heavier than normal nuclei, have velocities of galaxy-bound systems, and would be deeply penetrating. The detector is based on a stack of scintillator and piezoelectric elements which can provide information on both the charge state and mass, with the additional timing information allowing to determine the speed of the particle, searching for particles with velocities of the order of galactic rotation speed (v ≲ 250 km/s). In this work, we describe the apparatus and its observational capabilities.

## 1. Introduction

Strange Quark Matter (SQM) particles (also named strangelets or nuclearites) are composed of up, down, and strange quarks. Numerous studies and models suggest that SQM may be the fundamental state of matter instead of the ordinary hadrons (protons and neutrons). This is due to the inclusion of strange quarks that can lead to a more stable state of matter thanks to the presence of a third Fermi energy level that lowers the energy per nucleon. Other slow, heavy particles include Q-Balls, lumps of fermionic exotic compact stars, Primordial Black Holes (PBHs), mirror matter, Fermi balls, etc. We will use the term of SQM to refer to all the various types of quark-density particles.

These particles could represent a part, or even all, of the non-baryonic dark matter inferred by cosmological studies, without requiring new fundamental fields beyond the Standard Model of particle physics (SQM production would not interfere with standard calculations of primordial nucleosynthesis because it would be formed previously).

If SQM is the most fundamental state of matter, it would imply that compact objects such as neutron stars could have complex internal structures, associated with different hadronic compositions and densities, and stars entirely composed of SQM could exist, as well as new classes of white dwarfs. Such compact objects may also undergo internal transitions associated with powerful transient astrophysical emissions.

In this paper, we describe SQM-ISS, a space-station based instrument devoted to the direct detection of the passage of slow, heavy particles in a very wide range of mass and charge state. This instrument is part of a project “*SQM-ISS, Search for Strange Quark Matter and nuclearites on board the International Space Station*”, proposed in response to a 2022 ESA “call for ideas” for the “Reserve pools of Science Activities for ISS: A SciSpacE Announcement of Opportunity”. The proposal was selected by ESA as‬ an “excellent” evaluation and at the moment is in pre-phase A, currently undergoing studies of optimization of the various elements. Phase A is expected to begin at the end of 2024 with a relatively fast instrument development that plans for delivery of the instrument to the Italian/European space agencies by the second half of 2027 for a launch around the end of 2027, beginning of 2028.

These particles are presumed to have interstellar origin and thus have a local galactic orbital speed, v≃ 250 km/s, although both higher and lower velocities (in case they are bound in the solar system or local interstellar medium) can also be detected.

The location in space allows for a direct sampling of the solar system and interstellar material, without the risk of interaction/absorption in the atmosphere, allowing access to a lower mass range. The International Space Station (ISS) also provides a very stable microgravity environment, without the ground seismic noise. Furthermore, it constitutes a unique platform, providing power, telecommand/telemetry and, most of all, the possibility to physically transfer very large amounts of data to Earth using USB solid state flash drives.

While the detailed interaction modes of such a new fundamental state of matter are still a subject of debate, these particles can be detected via their ionization and/or emission of phonon/vibration in matter. In any event, the detection of a signal that is consistent with non-relativistic speeds would be an unambiguous signature of a very dense, so far undetected particle. In the absence of such a signal, we will significantly increase the existing constraints on the flux of such particles (under the most standard assumptions regarding their interactions), also targeting a wider range of potential candidates and extending the range of explored masses, thereby constraining their relevance as dark matter candidates and/or their interaction properties.

The detector is composed of a stack of scintillators (to detect the passage of SQM) and a stack of metal plates (to read with piezoelectric sensors the vibration caused by the passage of SQM). This innovative design allows to access both the energy deposition and the momentum deposition in the detector. The signals from the detectors are also read by a Time-of-Flight (ToF) system capable of measuring the speed of the particle. As said, these heavy particles are expected to be dense enough to cross the detector (unhindered by the ISS hull or the detector itself) at a speed consistent with the galactic orbital velocity of about 250 km/s.

## 2. Scientific Goals

### 2.1. Strange Quark Matter

The existence of a different state of hadronic matter other than the ordinary nuclear matter, called Strange Quark Matter (SQM), was proposed for the first time in the 1980s [1,2].

Strangelet mass can range from a minimum stable mass of ≃10s *A* [3], to values of a compact star $A≃10^{57}$ [4,5].

It can be produced just after the Big Bang [3], be part of baryonic dark matter [6], be present in the core of neutron stars, or exist as “strange quark stars”, either “pure” [4,7] or constituted of hadrons and quarks [8,9,10], and accelerated in the ergosphere of black holes [11] or in collisions between binary neutron stars [12,13,14], reaching the Earth [15].

### 2.2. Previous and Current Searches for Strange Quark Matter

Several experiments on ground, on balloons, and on satellites have searched for SQM, employing different detection techniques working under different hypotheses of their nature and means of interaction. A review of strangelet searches and models can be found in [16].

Heavy-ion experiments, such as the NA52 experiment at CERN [17], tried to produce long-lived massive strangelets in the hot and dense environment provided by two colliding nuclei, but no candidates were observed [18]. The Yale Wright Nuclear Structure Laboratory accelerator [19] was employed as a mass spectrometer to search for SQM particles in lunar soil, where they could have accumulated on the moon’s surface without being deflected by the geomagnetic field. No events were found, resulting in a concentration of strangelets in lunar soil estimated to be lower than $10^{-16}$ with respect to normal matter at 95% C.L (charge 5≤Z≤9 and *Z* = 11 and mass 42 < *A* < 70).

Such ground-based searches have the advantage of using a large amount of matter, providing high statistics. Using space-borne instruments or stratospheric balloon payloads, a direct search of SQM can also be performed, with the advantage of allowing the direct identification of these particles without specific assumptions of the interaction model with Earth’s atmosphere. Furthermore, it allows probing for lighter and potentially more abundant particles. Some relevant experiments are as follows:

- The balloon experiment HECRO-81 [20], which has reported the observation of two events with Z≃14. The mass number for these events was estimated to be A≃350.
- The ARIEL-6 satellite [21], with Cherenkov counters, which presented an analysis of *Z* ≥ 34 during 427 days of data taking, finding no SQM candidates.
- The HEAO-3 satellite [22] reported abundances of both odd–even element pairs (33 ≤ *Z* ≤ 60) and element groups (*Z* > 60) and did not find any candidate. The satellite had a geometrical factor of 413 cm^2^ sr; if we assume the same data set of [23] (between 17 October 1979 and 12 June 1980), we can estimate a limit of 7.8 × 10^9^ cm^2^ sr s.
- The SkyLab experiment [24], with 1.2 m^2^ Lexan track detectors, which did not find any valid candidate in the superheavy (*Z* > 110) nuclei range.
- The experiment TREK [25], which explored the *Z* > 50 region, finding no strangelet candidate.
- The BESS balloon spectrometer [26], whose searches yielded no candidates for 5 ≤ *Z* ≤ 26 and Z/A < 0.2.
- The AMS-01 experiment, which reported the observation of two events: *Z* = 8, *A* = 20, 3.93 GV and *Z* = 4, *A* = 50, 5.13 GV [16]. This is probably due to background since the following, much longer, AMS-02 mission found no events and lowered the limit on the flux of *Z* = 2 particles to ≲30 p/m^2^/s/sr [27].
- The PAMELA magnetic spectrometer, which searched for anomalous Z/A ratio (high atomic number *A* and low charge *Z*) particles in cosmic rays, using its Time-of-Flight system and permanent magnet spectrometer. The spectrometer explored the rigidity range between 1 and 1000 GV without finding traces of such particles, thereby setting an upper limit on the monochromatic strangelet flux in cosmic rays for particles having charge 1≤Z≤8 and baryonic mass $4\leq A\leq 1.2\times 10^{5}$ [28].
- Pi of the SKY, which searches for fast meteors in the sky [29].
- The DIMS experiment, which uses two cameras for stereoscopic view of meteors and determination of their trajectory [30].
- Mini-EUSO, which looks for fast meteor-like events from the ISS [31,32].

### 2.3. Primordial Black Holes and Fuzzballs

We now briefly discuss the possibility of detecting long-lived Primordial Black Holes (PBHs) [33,34] or other massive ‘Exotic’ Compact Objects (ECOs), such as fuzzballs, predicted by string theory or other extensions of General Relativity. Black hole (BH) mergers observed through their Gravitational Wave (GW) signals detected by LIGO/Virgo [35] have shown events with masses, spins and coalescence rates outside the expected range for stellar collapse but compatible with PBHs produced in the early universe [36]. Typical formation mechanisms require over-densities to collapse into PBHs after reentering the cosmological horizon in inflationary scenarios [37]. Other (non-)inflationary mechanisms require cosmic strings, domain walls, or phase transitions/crossovers [38], that naturally induce peaks in the PBH mass function around planetary mass, a solar mass, a few ten solar masses, and around a million solar masses [39]. Under suitable conditions, ‘standard’ confining theories like QCD (Quantum ChromoDynamics) have been recently argued to produce PBHs in the mass range from $10^{-17}$ to 10 solar masses [40].

String-theoretic implementations using D-brane models or highly excited strings with mass $M_{s}$∼10^17^ GeV and small/perturbative coupling $g_{s}$∼1/100 look viable in producing long-lived (horizon-less non-singular) fuzzballs with long-distance properties similar to PBHs [11]. PBHs or their ‘cousins’ (fuzzballs) may help in explaining the relation between the mass of a galaxy and that of its central black hole. Yet, for PBHs to be a viable candidate for a fraction of dark matter requires their Hawking evaporation to take longer than the age of the universe [41]; this constrains their masses to be $M_{\mathrm{PBH}}>10^{4}$ g. SQM-ISS can access this mass range and help put constraints on the abundance of PBHs or fuzzballs in a complementary way to other experiments, such as NANOGrav [42] that searches for an isotropic stochastic gravitational-wave background or those based on micro-lensing towards the galactic bulge or by quasars or on correlations between source-subtracted X-ray and CMB (Cosmic Microwave Background) fluctuations.

For their similarity with (charged) ‘Elementary BHs’ [43,44], we would also like to comment on Daemons (Dark Electric Matter Objects) [45] that have been proposed as the dark matter candidate and consist of charged (Ze∼10*e*) Planckian mass ($10^{19–22}$ GeV) particles, that can be also detected as catalysts of light–nuclei fusion [46].

### 2.4. Other Forms of Exotic Matter

**Figure 1:**
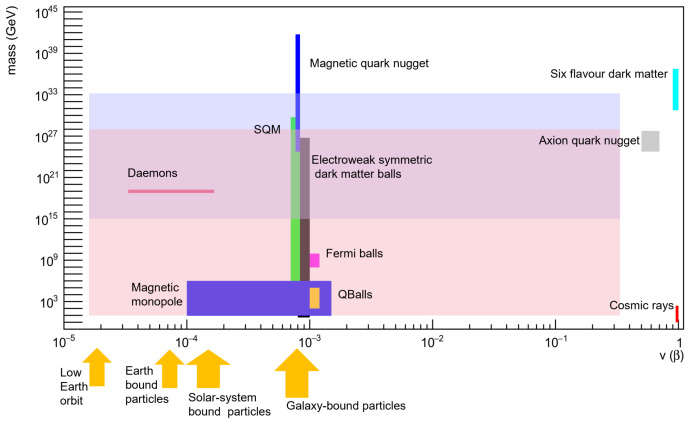
Plot of velocity and mass range for various hypothetical slow moving, massive particles. At the bottom right of the plot, we have cosmic ray nuclei with β≃1 (lower energy particles would be stopped by the hull of the ISS) and mass ≲200 GeV. Most of these hypothetical particle types have $\beta ≃7–8\times 10^{-4}$ since they are usually considered to be bound in our galaxy. However many of these particles can also have lower velocity since they could be bound in the local interstellar medium, in the vicinity of the solar system or even of our planet (e.g., Daemons). The masses range from the relatively light magnetic monopoles and Q-Balls to the heavier Fermi and dark matter balls. SQM and magnetic quark nuggets can reach the mass of a star, but only lighter fragments are expected to reach Earth. The expected ranges for velocities, masses and mass densities of these different candidates remain hypothetical with wide ranges acceptable for these values. This variety and uncertainties are an additional motivation to design an instrument sensitive to a wide range of masses, using complementary means of detection. In light pink, we show the mass–velocity range accessible by the scintillator/SiPM detectors and in light blue, the range of the piezoelectric detectors. The main target are thus very dense particles that would move at typical galactic orbital velocities, in the velocity range between $3\times 10^{-5}$ and $3\times 10^{-1}$ c.

Furthermore, there are many other possible massive compact objects that could be present in our galaxy: among them, we quote Q-Balls [47], magnetic monopoles [48], lumps of fermionic exotic compact stars [49], mirror matter [50], Fermi balls [51], electroweak symmetric dark matter balls [52], antiquark nuggets [53], axion quark nuggets [54], six-flavor quark matter [55], and non-strange quark matter [56]. The presumed mass and velocity range for the various candidates is shown in Figure 1.

We draw attention to the fact that the expected ranges for the velocities, masses, and mass densities of these different candidates remain hypothetical, and the literature does not offer precise values for any of these parameters. This variety and uncertainties are an additional motivation to design a detector sensitive to a wide range of masses, using complementary means of detection.

### 2.5. SQM as a Dark Matter Candidate

Many of these particles are also dark matter (DM) candidates: with the lack of evidence for supersymmetric neutralino-like particles from space-borne, underground-located or accelerator (e.g., LHC) experiments, these hypotheses are being examined with increasing detail. These particles could account for a part or all DM components in our galaxy. Therefore, the discovery of these particles would have repercussions not only in astrophysics and nuclear physics but also for the puzzle of the DM component in our universe. See [57] for a recent review of SQM as a DM candidate.

## 3. Observational Goals

SQM-ISS will perform a systematic search for SQM and other slow-moving massive particles. The dual system (scintillators and piezoelectric sensors) allows for a search in a very wide mass range: the SiPM-read plastic scintillators provide a signal proportional to the charge state of SQM mediated by the electromagnetic interaction, whereas piezoelectric detectors provide a signal that is proportional to the momentum of the particle. Since the nuclei of the metal are displaced by the SQM particle passage, the interaction can occur via the electromagnetic interaction (in the case of charged particles) and via the strong interaction (this is the only interaction possible in the case of neutral particles). The response of piezoelectric detectors allows to probe a very wide mass range $m>10^{-9}$ g that could also have a high charge that would saturate the SiPM response (there are two regimes depending on whether the particle is larger or smaller than the interatomic distance of the metal (e.g., 0.36 nm for Cu and 0.31 nm for W): in the former case, the signal is expected to be significantly higher than in the latter).

**Figure 2:**
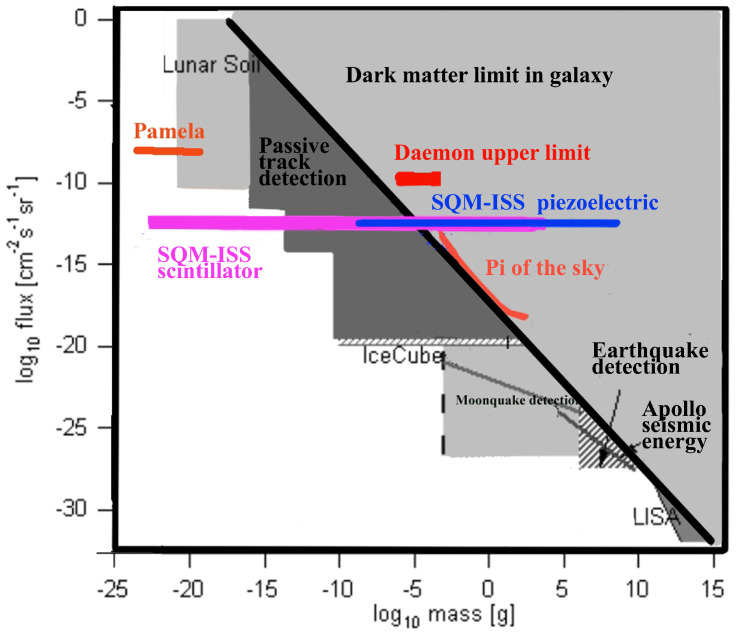
Current status of upper limits to SQM search as a function of the presumed mass. The particle mass can range from ≃10^2–3^ atomic nuclei to several tons. The diagonal black line is a limit posed by the amount of DM in the galaxy assuming that all DM is composed of SQM particles of just one mass. It is, however, possible (and indeed more probable) that (1) only part of DM is composed of SQM; (2) the particles have a very wide range of masses; and (3) speed may be non-galactic but rather solar system bound (<72 km/s) or local interstellar medium bound. Furthermore, the various upper limits shown here were obtained with various means, e.g., lunar soil with mass spectrometer, track presence observing mica and Skylab-Lexan track detector (or rather the lack of them), Apollo seismograph observing earthquakes generated on the moon by falling meteors, etc. Thus, depending on the kind of particle considered (e.g., Daemons [45,58]), some upper limits may not apply since the detection method would not have been able to observe them. Also, if the speed is below galactic speed, the interaction energy and the mass would be skewed. The horizontal bars for SQM-ISS refer to an upper limit after about three years of observations and show the different mass range for scintillators and piezoelectric detectors.

The current status of upper limits to SQM search is presented in Figure 2 together with the goals of SQM-ISS. Some exclusion zones shown are not firmly established, as the various measurements assume different modes of interactions. The parameter space that has already been explored and supposedly excluded might be more multi-faceted than what is presented here for simplicity. In Figure 2, we show also the expected upper limit of our detector with respect to the current one [45] for the Daemons class of particles.

The experiment aims to detect a wide range of different hypothetical compact and dense objects that would be much heavier than normal nuclei, have velocities of galaxy-bound systems and would be deeply penetrating. The lower range of velocities (≲90 km/s) is particularly interesting since there are various scenarios in which these particles are bound in the solar system on Strongly Elongated Earth Crossing Heliocentric Orbits (SEECHOs) or even in the gravitational field of the Near Earth Almost Circular Heliocentric Orbits (NEACHOs) and Geocentric Earth Surface Crossing Orbits (GESCOs) [45,58].

As discussed, most models for SQM and other heavy objects assume that these particles are gravitationally bound to our galaxy and have a speed up to 250 km/s, thus having a time of flight between the scintillators of about 0.3 μs in 7 cm (the distance between the first and last scintillator planes in the detector; see Section 4). This time interval is about three orders of magnitude lower than that of cosmic rays of galactic, solar, or trapped origin, which must have relativistic speed (0.23 ns crossing time in 7 cm) to cross the hull of the ISS and the detector planes. Indeed, cosmic rays with speeds of a few hundred or thousand km/s, typical of solar wind, are deflected by the geomagnetic field; those that manage to reach the ISS would be stopped by the hull of the station and the detector container. We estimate that a proton would need to have at least a velocity of ≃0.4 c = 120,000 km/s (1.02 GeV) to trigger the system, but in that case, it would be rejected by the ToF signal. This allows to have a very low particle background, mostly due to the random coincidence of two independent events, which can be calculated and directly measured in situ.

## 4. Detector Description

**Figure 3:**
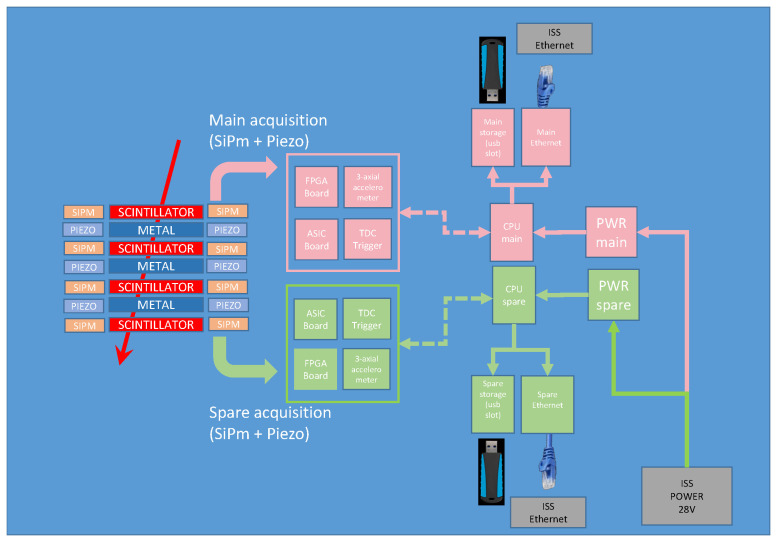
Block scheme of the elements present in SQM-ISS: a stack of plastic scintillators and metal plates are read out by SiPMs and piezoelectric detectors, respectively. The signals are acquired by the front-end electronics (ASIC), and both amplitude (ADC) and time (TDC) signals are saved. This information is used by the trigger FPGA to decide whether to store the event. If it passes the trigger criteria, data are stored by the onboard CPU on USB solid state flash drive and sent via telemetry to ground.

A block scheme of the instrument is shown in Figure 3. The sensitive part of the detector is composed of a stack of four scintillators and three metal planes, all having 10×10×0.5 cm^3^ size. The overall detector height is 7 cm. The geometrical factor of a four-fold coincidence of the scintillator planes is 111 cm^2^ sr, while a three-fold coincidence of either the scintillators or the metal plates results in a geometrical factor of 154 cm^2^ sr. The whole system is housed in a 15×15×14 cm^3^ aluminum container (no sharp edges, thickness 2 mm) and weighs ≃10 kg.

Signals from the scintillators are acquired from both sides by SiPMs and those of the metal plates by piezoelectric sensors. All signals are digitized by a front-end ASIC (Application Specific Integrated Circuit) and sent to an FPGA for data handling and triggering. The candidate events passing the selection criteria are then stored by the CPU for transmission to the ground (a subset of data) and permanent storage on the USB solid state flash drive.

**Figure 4:**
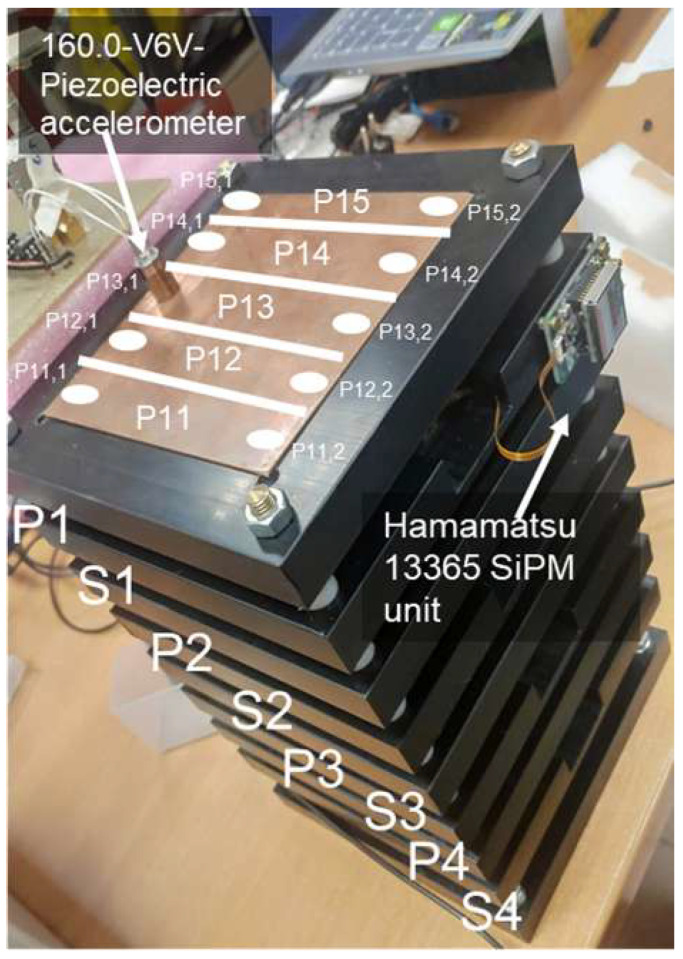
Picture of the tower of metal and scintillator elements used in the laboratory model of the instrument. The sensitive elements have an overall area of 10×10 cm^2^. Each detector plane is divided into five strips of 2 cm × 10 cm each. Note that this model has one additional piezoelectric plane with respect to the scheme of Figure 3. The white lines show the planned division of the first plane in five strips, read by a piezoelectric detector at each of the two extremities. The same division is present in the scintillator planes.

A picture of the laboratory model of the instrument with the detector stack is shown in Figure 4.

### 4.1. Detectors

**Figure 5:**
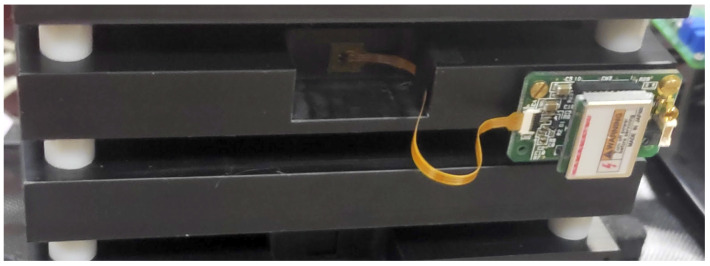
Close-up of the Hamamatsu 1663 SiPM (Hamamatsu Photonics K.K., Shizuoka, Japan) module located on the detector tower. In the center of the picture, connected with a kapton flex cable, is the SiPM (3 × 3 mm^2^) and the temperature sensor. The electronic unit, containing the power supply, the temperature compensation circuit, and the pre-amplification stage, is located on the right of the picture. The size of the electronic unit is 36 × 22 mm^2^.

The EJ-228 plastic scintillator (SCIONIX Holland B.V.) is used as a detector for charged particles. It is a polyvinyltoluene, fast scintillator (rise time 0.5 ns, decay time 1.4 ns) with an emission peak at 391 nm wavelenght. The SiPMs are directly coupled (with optical grease) to the scintillators and connected with a kapton flex cable to the Hamamatsu 13365-3050 module (Hamamatsu Photonics K.K., Shizuoka, Japan) (see Figure 5), a single-channel unit which provides signal pre-amplification, high-voltage (70 V) power supply, and a temperature compensation circuit which varies the high-voltage value according to temperature fluctuations to keep the SiPM gain constant. The photosensitive area of the SiPM is 3 × 3 mm^2^, with an analog output. The unit requires ±5 V power supply to operate. Each scintillator plane is divided in five strips (2.0 cm × 10 cm) to improve the timing resolution. Each strip is read by two SiPMs (one per side), for a total of 10 SiPMs for each plane. Four scintillator planes are present in the SQM-ISS detector.

**Figure 6:**
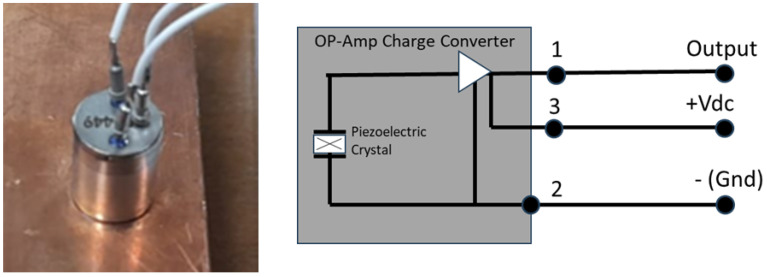
**Left**: Picture of the piezoelectric detector glued to a copper metal slab. **Right**: Scheme of the piezoelectric detector unit and its electrical configuration. Vibrations on the metal plate are transmitted to the crystal, producing an electric signal which is pre-amplified inside the unit.

Copper plates are used to detect vibrations coming from the passage of heavy particles. The vibrations are read by piezoelectric detectors (160.01-V6V-1 units, produced by DSPM Industria, Milan, Italy). It is an accelerometer with a three-wire configuration (see Figure 6), 100 mV/g ± 20% sensitivity. The digitized waveforms from the piezoelectric detectors are read out by the ASIC and thresholded to produce the logic signal for the triggering conditions and for the ToF readout. Also in this case, each plane is divided into five strips (2.0 cm × 10 cm) to improve the timing resolution. Each strip is read by two piezoelectric detectors (one per side), for a total of 10 in each plane. Three metal planes are present in the detector. A study to improve the sensitivity of the instrument changing the metal material, the thickness of the plate, and the coupling to the vertical columns that hold the detector tower together is also underway.

### 4.2. Trigger

The two systems operate independently and both provide charge (via an ADC, Analog-to-Digital Converter) and time (via a TDC, Time-to-Digital Converter) information once the signals are read by the front-end ASIC. A single signature from either of the detectors would be enough to identify SQM, although, naturally, a joint signal would provide much stronger evidence and more details on the nature of the particle.

The coincidence between signals from different planes provides the trigger for the acquisition. To avoid random coincidences due to crossing protons, an high threshold (corresponding to the release of Z > 4 nuclei at relativistic speed, and thus, ≃16 mip) will be placed. The threshold can be changed from ground via telecommand. In this way, the expected rate from coincidences will be very low (a few events per hour), although the detector should be able to handle a few Hz of trigger rate (the bottleneck being the telemetry to send data to the ground). Different triggers are foreseen with different priorities:

(a) Relativistic charged nuclei with Z≥4. They are expected to hit only one strip in each scintillator plane and provide no detectable signal in the piezoelectric detector. Crossing time is $t_{cross}≃0.2$ ns. The high-charge threshold is placed in order to exclude random coincidences from protons and helium nuclei.
(b) Slow charged particles with a signal present only in one strip in each plane of the scintillators. With a threshold of v≤1000 km/s, the crossing time is expected to be t≥70 ns. At this speed, the range of any ordinary nucleus would be lower than the thickness of the hull of the station.
(c) Slow particles providing a signal only in the metal plates. This trigger could occur in the case of neutral SQM or macro-particles that do not ionize the scintillators but excite the nuclei of the metal planes.
(d) Slow charged particles providing a signal in both the scintillator and metal plates. These would be the golden candidates for SQM particles.
(e) Shower events at relativistic speed, leaving a signal with equivalent charge $Z^{2}\geq 16$ in one or more strips of each plane of the detector.

All events of type (b, c, and d) represent the signal, whereas types (a) and (e) represent the background. After acquisition and processing, data are saved on a USB solid state flash drive (each of 1–2 Tbyte size) and sent to the ground via telemetry. Most of the background triggers are only saved on a USB flash drive to reduce the burden on the telemetry to the ground.

### 4.3. Time of Flight System

Given the relatively low speed of the particles we are looking for, the gate for the ToF system has to stay open for a time ≃ 2.5 μs (for a particle crossing, the detector with a speed of 28 km/s, and 25 μs for a particle with a speed of 2.8 km/s). To avoid random coincidences due to the passage of different relativistic protons (especially in the South Atlantic Anomaly (SAA) region, where the International Space Station crosses the inner Van Allen Belt), a threshold of charge Z≥4 is used. This also excludes signals coming from the low-energy protons stopping in the detector. The threshold and the duration of the gate can be configured by ground commands. To increase the geometrical factor of the detector, the trigger accepts also triple coincidences from the scintillator planes:

(1) $$\left(\left(S_{1}\times S_{2}\times S_{3}\right)+\left(S_{2}\times S_{3}\times S_{4}\right)\right)$$

with particles coming from both directions:

(2) $$\left(\left(T_{1}<T_{2}<T_{3}\right)+\left(T_{3}<T_{2}<T_{1}\right)\right)$$

where $S_{i}$ and $T_{i}$ stand for the ADC and TDC signal coming from a strip hit on plane *i*.

Assuming only one strip hit per plane, the time of arrival on plane *i* is the sum of the times of arrival on each extremity of scintillator strip *j*:

(3) $$T_{i}=T_{ij,1}+T_{ij,2}$$

so that, for instance, the speed calculated between plane 1 and 4 is:

(4) $$V_{14}=D_{14}/\left(T_{13,1}+T_{13,2}-T_{42,1}-T_{42,2}\right)$$

assuming—for instance—that the particle hit strip 3 of plane 1 and strip 2 of plane 4 and $D_{14}$ is the distance between the two planes. The sum of the two arrival times on each strip has the advantage of being invariant from the impact point of the particle. Assuming that the particle hits the strip at distance *x* at time $t_{0}$ from detector 1, we have:

(5) $$\begin{array}{c} T_{ij,1}=t_{0}+x/c \end{array}$$

(6) $$\begin{array}{c} T_{ij,2}=t_{0}+\left(L-x\right)/c \end{array}$$

(7) $$\begin{array}{c} T_{ij,1}+T_{ij,2}=L/c+2\cdot t_{0} \end{array}$$

where *L* is the strip length, and *c* is the speed of light.

The position *x* on the strip can be determined by the difference of the arrival times at the extremities:

(8) $$L-2x=c\cdot (T_{ij,2}-T_{ij,1})$$

The “differences of the sum” technique has the advantage of providing a time of arrival on each plane that is independent of the impact point on the strip. This is particularly useful in the case of the timing of the signals coming from the piezoelectric detectors, where the speed of propagation of the vibrations in the metal (≃3 km/s) can be slower or comparable to the speed of the SQM particle.

### 4.4. ISS Interfaces

The only connections to the ISS are power, ground, and telemetry (including timing information). The power connection is via 28 V, and the power consumption is <25 W. The detector will be attached by its bottom side via velcro or straps (soft coupling) to any wall or behind the panels of the station (hard coupling is not recommended since it would transmit more parasitic vibrations from the ISS to the piezoelectric detectors). To minimize the impact to the crew, one of the less busy modules can be employed, with the field of view of the telescope orthogonal to the longitudinal axis of the module in order to minimize the passive material. After inserting the USB solid state flash drive, connecting ground, power and ethernet connectors to the detector, the instrument can be turned on and work in standalone mode for several weeks: the link to the ISS network and downlink to Earth would allow for remote monitoring without the need for crew time. Connection to the ISS network also provides the timing of the instrument (≃500 ms precision) and is used to receive telecommands from ground.

## 5. Conclusions and Future Work

In this work, we have described SQM-ISS, an instrument devoted to the search for SQM and other types of exotic, slow moving particles in space. The detector will employ a dual detection system, with scintillators and piezoelectric sensors, read out by an ADC for charge information and a TDC for timing information, allowing to look for signatures coming from slow, deeply penetrating particles. The project has been submitted in response to the ESA “call for ideas” for the “Reserve pools of Science Activities for ISS: A SciSpacE Announcement of Opportunity” and has been selected in 2023 with an “excellent” score. At the moment, the instrument is in pre-phase A, and is currently undergoing studies of optimization of various elements. Phase A is expected to begin at the end of 2024 with a relatively fast instrument development that plans for the delivery of the instrument to the Italian/European space agencies by the second half of 2027 for a launch around the end of 2027, beginning of 2028.

## Data Availability

Data are contained within the article.
